# A scalable sparse neural network framework for rare cell type annotation of single-cell transcriptome data

**DOI:** 10.1038/s42003-023-04928-6

**Published:** 2023-05-20

**Authors:** Yuqi Cheng, Xingyu Fan, Jianing Zhang, Yu Li

**Affiliations:** 1 Department of Computer Science and Engineering (CSE), The Chinese University of Hong Kong (CUHK), Hong Kong SAR, China; 2 School of Computational Science and Engineering, Georgia Institute of Technology, Atlanta, GA USA; 3 School of Information and Software Engineering, University of Electronic Science and Technology of China, 610054 Chengdu, China; 4 The CUHK Shenzhen Research Institute, Hi-Tech Park, Nanshan, 518057 Shenzhen, China

**Keywords:** Computational models, Data integration

## Abstract

Automatic cell type annotation methods are increasingly used in single-cell RNA sequencing (scRNA-seq) analysis due to their fast and precise advantages. However, current methods often fail to account for the imbalance of scRNA-seq datasets and ignore information from smaller populations, leading to significant biological analysis errors. Here, we introduce scBalance, an integrated sparse neural network framework that incorporates adaptive weight sampling and dropout techniques for auto-annotation tasks. Using 20 scRNA-seq datasets with varying scales and degrees of imbalance, we demonstrate that scBalance outperforms current methods in both intra- and inter-dataset annotation tasks. Additionally, scBalance displays impressive scalability in identifying rare cell types in million-level datasets, as shown in the bronchoalveolar cell landscape. scBalance is also significantly faster than commonly used tools and comes in a user-friendly format, making it a superior tool for scRNA-seq analysis on the Python-based platform.

## Abstract (web summary)

An integrated sparse neural network framework scBalance incorporates adaptive weight sampling and dropout techniques to handle noise and imbalanced datasets and achieves high classification accuracy for both rare and major cell types.

## Introduction

Since the first establishment of single-cell RNA sequencing (scRNA-seq) by Tang et al. in 2009^1^, this technology has rapidly become popular among scientists in various biological research fields. Compared with traditional bulk RNA sequencing which only measures the average gene expression level of the samples, scRNA-seq provides a powerful method to profile transcriptomes on the cell-specific level. Therefore, it could enable analyzing individual cells and give a more informative insight into cell heterogeneity. The development of scRNA-seq technology has been widely used in several biological research areas, such as cancer research^2,3^, COVID analysis^4,5^, developmental biology research^6^, etc. In these studies, uncovering and identifying cellular populations is one of the most critical tasks.

Typically, cell-type annotation involves two steps: (1) clustering cells into different subgroups and (2) labeling each group with a specific type manually based on the prior-known marker genes. A number of unsupervised machine-learning algorithms have been developed, including classical machine-learning-based methods such as Seurat^7^ and Scanpy^8^, and newly published deep learning-based methods, such as scDHA^9^ and CLEAR^10^. However, these methods can be time-consuming and burdensome. For those who do not have too much knowledge of the marker genes, this approach could cost far more time than expected. Automatic cell-type annotation methods, in contrast, do not suffer from the manual labeling process. Different from the unsupervised methods, automatic cell-type identification tools are mainly designed based on supervised learning frameworks. Taking advantage of its fast and precise features, they are becoming predominant tools to identify cell types in single-cell experiments. With the unprecedented boom in the well-annotated scRNA-seq atlas and the rapid promotion of the Human Cell Atlas project^11,12^, auto-annotation tools are facing a more broad prospect than anytime before. Up to now, 32 auto-annotation tools are developed and published^13^. For example, SingleCellNet^14^ utilizes a random-forest classifier to solve the cross-platform and cross-species annotation tasks. ACTINN^15^ implements a simple artificial neural network to overcome the batch effect.

While numerous tools have been established in recent years, most of those often fail to identify the entire population because of the existence of rare cell types. From the perspective of cell composition, scRNA-seq datasets are always imbalanced, which have common cell types and rare cell types. The rare population is a small proportion of cells in the single-cell dataset. For example, the dendritic cell usually takes 1–5% of peripheral blood mononuclear cells (PBMCs), especially in large datasets^16,17^. When we train an auto-annotation tool, the classifier is consistently unable to learn their information thus hard to identify these cell types in the query dataset. However, these rare populations can be crucial, especially in disease research^18^. Recently, some cluster detection methods have noticed this point^19,20^ but few classification methods focused on the cell population imbalance. Meanwhile, we also find that the existing methods have two other main deficiencies. (1) Lack of scalability. Recent scRNA-seq experimental platforms enable investigations of million-level cells^21,22^. Notably, one of the most recent COVID PBMC atlas has reached 1.5 million cells^17^. Thus computation speed restriction will render auto-annotation packages poorly scalable for the million-level dataset. Moreover, large-scale reference datasets add more challenges for learning rare cell types in classifier training, which leads current software more difficult to identify minor groups. Most recently published paper has elevated the training scale to 600 K cells^23^, however, no published tools successfully report scalability on the million-level cell atlas. (2) Compatibility of the existing tools is not as good as expected. Among the existing Python-based tools, most of the tools such as ACTINN^15^, scPretrain^24^, scCapNet^25^, and MarkerCount^26^ are script-based. Considering that Seurat and Scanpy are both packages that can be downloaded from a standard software repository (e.g., PyPI), running an external Python script on the server will add an additional burden to the user. In addition, some of the tools are no longer maintained or are not able to use. All these challenges together make a new annotation tool that has a balanced ability to label major and minor cell types in a scalable manner become necessary.

Here, we introduce scBalance, a sparse neural network framework that can automatically label rare cell types in scRNA-seq datasets of all scales. scBalance leverages the combination of weight sampling and sparse neural network, whereby minor (rare) cell types are more informative without harming the annotation efficiency of the common (major) cell populations. We evaluated scBalance on real datasets with varying degrees of cell population imbalance and scale on both intra- and inter-dataset annotation tasks, and compared its performance to popular published tools such as Scmap-cell^27^, Scmap-cluster^27^, SingleCellNet^14^, SingleR^28^, scVI^29^, scPred^30^, and MARS^31^. Each method represents a traditional machine-learning algorithm such as Scmap-cell is based on KNN, SingleCellNet is based on Random Forest and scVI and MARS are deep learning-based methods. Among them, our method consistently outperformed these tools in identifying rare cell types, while maintaining high accuracy in annotating major cell types. Additionally, scBalance also demonstrated fast and stable computation speeds outperforming other approaches across all dataset sizes. Moreover, scBalance was successfully trained on a published COVID immune cell atlas^17^ (1.5 million cells) and further annotated and discovered new cell types in the published bronchoalveolar lavage fluid (BALF) scRNA-seq dataset^32^. Satisfyingly, our method identified more rare cell types than the original analysis. Our user-friendly application is compatible with Scanpy and Anndata, and can be easily downloaded from PyPI and used as an external API of Scanpy (https://github.com/yuqcheng/scBalance).

## Results

### Overview of the architecture of scBalance

**Fig. 1:**
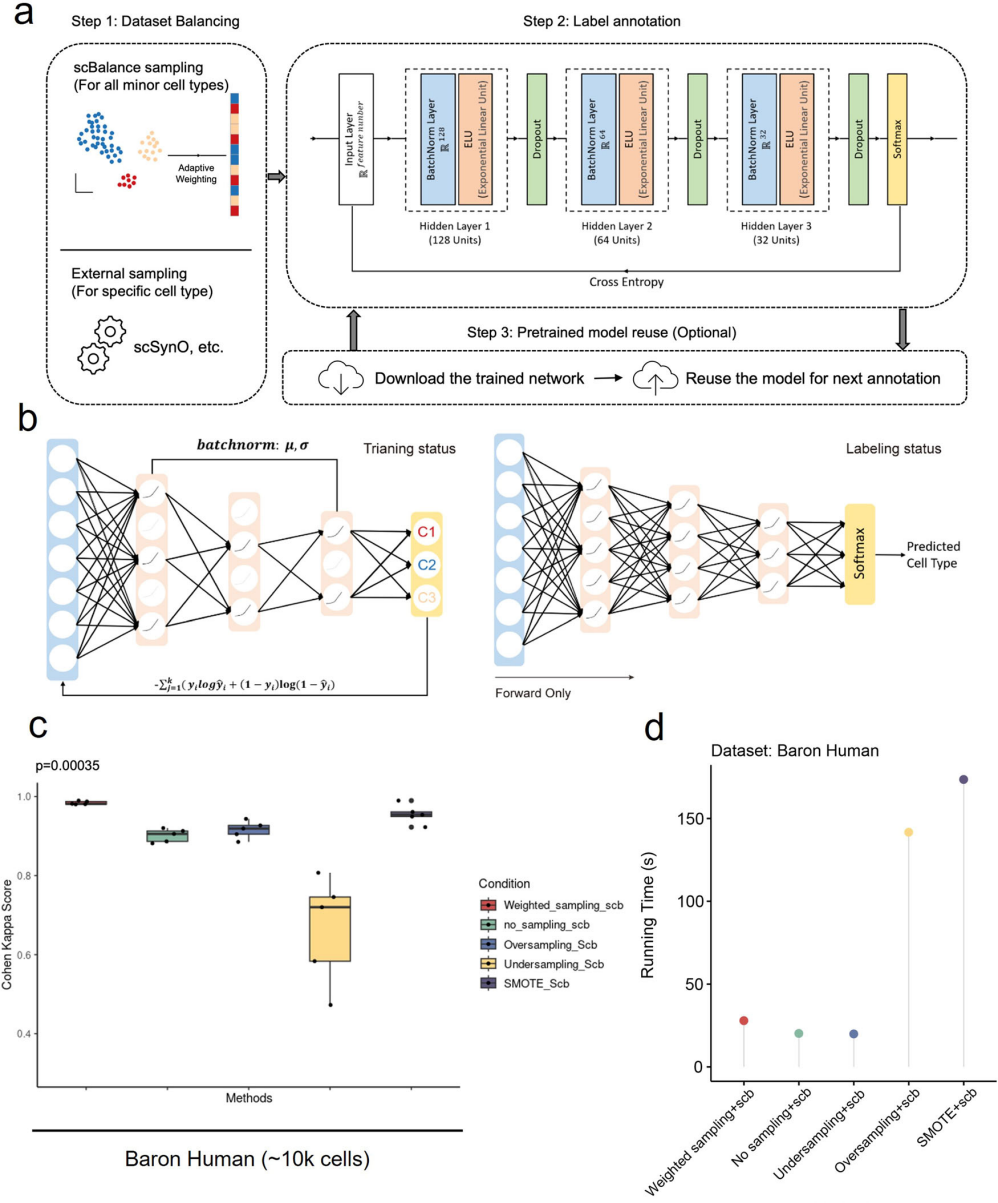
Schematic overview of scBalance. **a** The method is constructed based on the supervised learning framework, which contains a dataset-balancing module and a dropout neural network module. Step 1 Upper: With our adaptive weighted sampling, scBalance will automatically choose the weight for each cell type in the reference dataset and construct the training batch. Lower: Users can choose an external dataset-balancing method, such as scSynO, instead of using our internal balancing method. Only the classifier will be used in this case. Step 2: While training, scBalance will iteratively learn mini batches from a three-layer neural network until the cross-entropy loss converges. **b** Dropout setting in different stages. In the training stage, scBalance randomly disables neurons in the network. The dropout layer is binary with a rate of 0.5. All the dropped units will be reconnected in the testing stage. The prediction will be processed by a fully connected neural network. **c** Evaluation of balancing methods shows that our sampling method outperforms simple oversampling and downsampling methods as well as the SMOTE method. The p-value is from a significance test of scBalance and SMOTE (*n* = 5 for each boxplot). **d** Comparison of running times among different sampling techniques.

scBalance provides an integrative deep learning framework to perform accurate and fast cell-type annotation, especially on rare cell types, in a scalable manner (Fig. 1). The structure of the scBalance includes two parts, a weight sampling technique that adapts to imbalanced scRNA-seq datasets, and a sparse neural network that efficiently annotates cell types.

First, different from all existing tools, we use a specially designed weight sampling technique to adaptively process the imbalanced scRNA-seq dataset. Unlike exsiting methods that use synthetic-based technique^33,34^, our method incorporates the balancing technique into training batches so that will not generate new points, thus can save memory space and speeding up training. This design is particularly useful for the atlas-scale dataset, where generating new dataset points is impractical. In scBalance, to keep as much information as possible and avoid a huge training time cost, we randomly over-sample the rare populations (minority classes) as well as under-sample the common cell types (majority classes) in each training batch (Fig. 1a, Step 1). The sampling process is done with replacement, and the sampling ratio is adaptive for different reference datasets, defined as the cell-type proportions of the true label provided by the reference set. This minimizes overfitting in the oversampling, thus maintaining a promising performance of the generalization ability of scBalance. Meanwhile, regarding the enormous overlapping expression information in the common populations, the under-sampling of the major class enables scBalance to use a relatively small training size with an abundance of training information. Leveraging this design, scBalance yields an exceptional performance in learning features of rare cell types as well as maintains a strong ability in classifying all major cell types, thus also improving its overall annotation accuracy. To testify to the performance of our internal sampling method, we benchmarked it with popularly used balancing techniques such as simple oversampling and downsampling as well as Synthetic Minority Over-sampling Technique (SMOTE). The results show that our internal balancing method improves classification accuracy compared with simple over- and downsampling and also outperforms the synthetic method SMOTE (Fig. 1c and Supplementary Fig. 1). Notably, our method provides a faster and space-saving balancing solution compared with normally used balancing methods (Fig. 1d and Supplementary Fig. 2a, b, and Supplementary Data 1). Because our method is coupled with the training process, it will not need to generate new data points, thus saving time and memory space. Additionally, scBalance also provides an interface for users who would like to explore specific minor cell types in a more detailed granularity. It allows datasets processed by external sampling methods such as scSynO^34^. In this case, only scBalance classifier will be used.

Moreover, we notice that the reference dataset and the prediction dataset can be generated by different sequencing platforms and protocols such as the 10X platform and Smart-seq platform, thus will naturally introduce different noises such as gene detection dropouts and random sequencing error^35^. To address this issue, scBalance considers random noise as a type of overfitting event and implements the dropout^36^ technique to mitigate this problem. The dropout layer, due to its excellent capacity of reducing overfitting, also enhances the learning ability of the scBalance to the resampled minor cell types. Additionally, scBalance provides a network reusing option for atlas-scale training scenario, enabling users to avoid the significant time cost of training the model again for the same dataset (Fig. 1a, Step 3).

Taken together, scBalance provides a three hidden layers network structure with a batchnorm and dropout setting in each layer. The activation function is set as an exponential linear unit (ELU)^37^ and the output layer uses Softmax. In the training mode (Fig. 1a, Step 2), units in the hidden layer are randomly disabled to help reduce the influence of noises on the training process. In the predicting mode, the network will be set as a fully connected status to keep all parameters being used in the forward process. The model evaluation and backpropagation are based on the cross-entropy loss function and Adam optimizer. To speed up the training and predicting process, scBalance also includes a graphics processing unit (GPU) mode which reduces the running time of the classifier by 25–30%. Overall, scBalance is well-designed to handle different types of noises and imbalanced datasets while achieving high classification accuracy for rare and major cell types.

### scBalance accurately identifies rare cell population in the intra-dataset labeling task

**Fig. 2:**
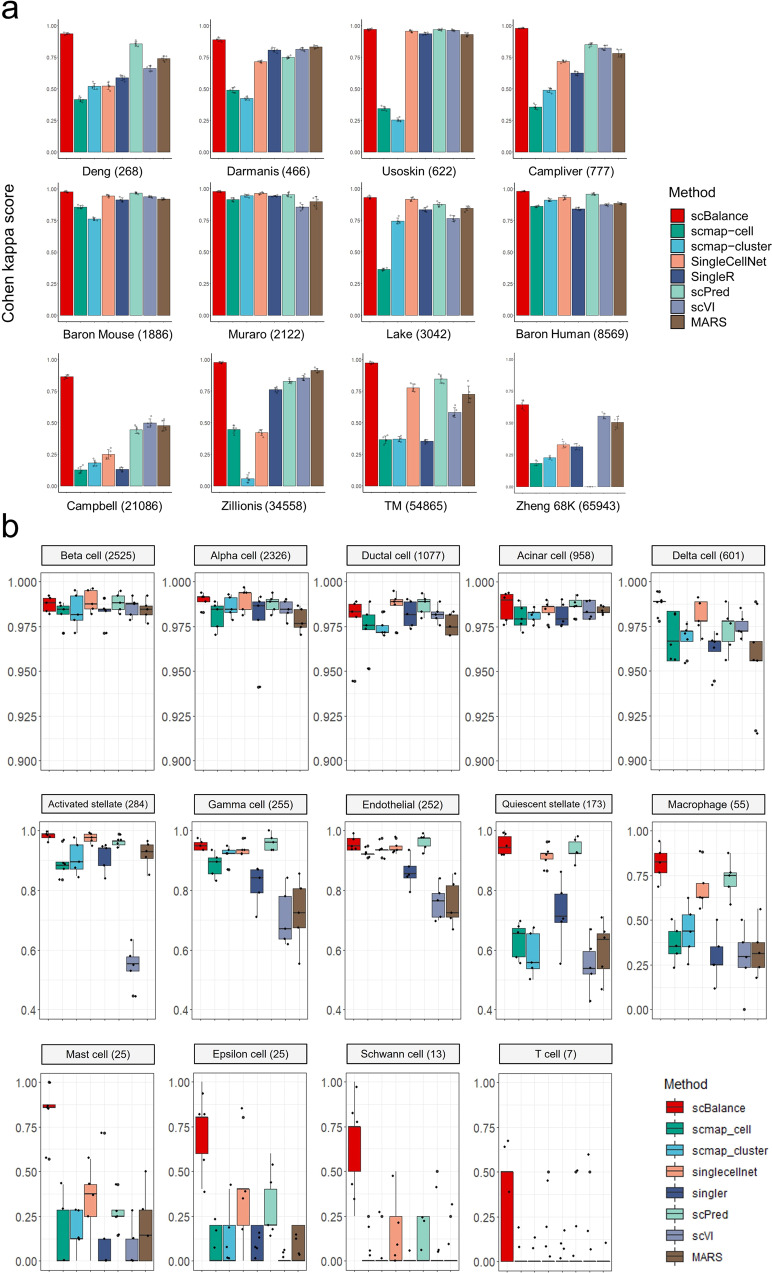
scBalance demonstrates superior performance in identifying rare cell populations on the intra-dataset annotation task. **a** Overall annotation performance, as measured by Cohen’s Kappa score, compared to existing methods on multiple datasets (cell number indicated after dataset name). scBalance consistently outperforms other methods in accurately identifying minor cell populations across all twelve datasets. (*n* = 5 for each barplot and error bar. Error bars are defined as mean value ±  standard deviation). **b** Cell-type-specific accuracy benchmarking on the Baron Human dataset (*n* = 5 for each boxplot). The number following each cell-type name represents the number of cells in that type. scBalance achieves higher accuracy in identifying rare cell types compared to other methods.

We first demonstrated the rare cell-type identification ability of scBalance in the baseline test. To evaluate performance, we used twelve scRNA-seq datasets with different imbalance degrees and different cell numbers, which were divided into train sets and test sets. To ensure a more comprehensive test, most of the datasets are generated from different sequencing platforms (see “Methods” and Table 1). The true label information of these datasets is only available in evaluating prediction results. Here, we compared scBalance with seven methods that are widely used for scRNA-seq cell-type identification: SingleCellNet^14^, SingleR^28^, scVI^29^, scmap-cell^27^, scmap-cluster^27^, scPred^30^ and MARS^31^, in which scPred and MARS also claimed the ability to treat imbalance single-cell dataset in their papers, and scVI and MARS are deep learning-based methods like scBalance. To ensure our benchmark comparison is under a fair experiment, we used a uniform preprocessing process for each tool and set all parameters as default. All the experiments were conducted based on the fivefold cross-validation to quantify the classification variability. Detailed protocol can be found in “Methods”. We used Cohen’s kappa score to quantitatively evaluate the performance of scBalance and the other seven methods (Fig. 2a). According to the result, scBalance outperforms all other methods on most of these twelve datasets by achieving the highest Cohen’s kappa score. Notably, scBalance particularly performs well on large and complex datasets such as Campbell and Zillions. And the performance of scBalance is the most stable among all these seven methods, giving it an advantage in further atlas-scale reference training. Because Cohen’s kappa score provides a minority class sensitive metric, outperforming on this score gives preliminary evidence that the scBalance has more advantages in rare population annotation.

**Table 1:** Description of the 23 datasets used in the experiments.

Application	Dataset	Cell number	Class number	Protocal	Reference	Minor cell type (<5% total cell number)	Cell number of the smallest cell type
	Deng	268	6	Smart-seq2	Deng et al.^49^	2	12
	Darmanis	466	9	SMARTer	Darmanis et al.^50^	3	16
	Usoskin	622	4	STRT-Seq	Usoskin et al.^51^	0	81
	CampLiver	777	7	SMARTer	Camp et al.^52^	0	70
	Baron Mouse	1886	13	inDrop	Baron et al.^53^	8	6
Intra-datast Benchmark	Muraro	2122	9	CEL-Seq2	Muraro et al.^54^	4	3
	Lake	3042	16	Fluidigm C1	Lake et al.^55^	10	45
	Baron Human	8569	14	inDrop	Baron et al.^53^	9	7
	Campbell	21,086	32	Drop-Seq	Campbell et al.^56^	29	30
	Zilionis	34,558	9	inDrop	Zilionis et al.^57^	4	108
	TM (Tabula Muris)	54,865	55	10X Genomics	Schaum et al.^58^	48	24
	Zheng 68 K	65,943	11	10X Genomics	Zheng et al.^59^	6	92
	PbmcBench 10X (V2)	23,154	9	10X Genomics (v2)	Ding et al.^60^	4	132
	PbmcBench 10X (V3)	19,690	8	10X Genomics (v3)	Ding et al.^60^	4	209
	PbmcBench (CEL-Seq)	19,754	7	CEL-Seq2	Ding et al.^60^	3	559
	PbmcBench (Drop-Seq)	23,154	9	Drop-Seq	Ding et al.^60^	4	102
	PbmcBench (inDrop)	21,832	7	inDrop	Ding et al.^60^	3	134
Inter-dataset Benchmark	PbmcBench (Seq-Well)	18,966	7	Seq-Well	Ding et al.^60^	3	102
	PbmcBench (SMARTseq)	18,886	6	SMART-Seq2	Ding et al.^60^	2	569
	Xin	1449	4	SMARTer	Xin et al.^61^	1	46
	Baron Human	8569	14	inDrop	Baron et al.^53^	9	7
	Segerstolpe	2133	13	SMART-s	Segerstolpe et al.^62^	7	5
	Muraro	2122	9	CEL-Seq	Muraro et al.^54^	4	3
Case study	Cardiac Atlas	487,106	11	10X Genomics	Litviňuková et al.^41^	5	3799
	PKU_Covid Atlas	1,462,702	64	10X Genomics	Ren et al.^17^	54	17

The Dataset column presents the dataset name we used in the article. The cell number column shows the total number of cells in the dataset before preprocessing. The protocol column shows the sequencing method that generates this dataset. The minor cell-type column shows the number of the cell types which has cells less than 5% of the total cell numbers. The cell number of the smallest cell-type column presents the number of cells in the cell population that has the smallest cell number. All the usages of the corresponding dataset are shown in the Application column.

To better demonstrate the ability of scBalance to accurately annotate minor cell populations, we further investigated the accuracy of each cell type to show whether the overall high performance is exactly obtained by the improvement of minor cell-type identification (Fig. 2b and Supplementary Figs. 2–4, and Supplementary Data 2). We categorized these datasets into three classes: (1) large datasets with a simple cell composition, such as Baron Human, Lake, and Zillions; (2) small datasets with a simple cell background, such as Muraro, Baron Mouse, Deng, etc.; and (3) datasets with complex cell structures, for example, Zheng 68 K, which is primarily composed of T cell and its subtypes so that cells are sharing a high similarity. We first analyzed the performance of scBalance on the Baron Human dataset (Fig. 2b and Supplementary Data 3) and found that all methods perform well on large populations, such as the Beta cell and Alpha cell. However, in minor cell types such as the Mast cell and Epsilon cell, the performance of scBalance still keeps stable and promising, while the other methods fail to recognize most of these rare cell types. These results demonstrate the ability of scBalance to annotate minor cell populations in regular datasets. Similar results can also be found in the result of the small dataset (Supplementary Fig. 3). Furthermore, we were also interested in the performance of scBalance on the dataset with a complex cell background. By analyzing the result on the Zheng 68 K dataset (Supplementary Fig. 4), we found that scBalance is still the best method for identifying rare cell types while maintaining high accuracy in the other types. This result further gives scBalance a practical advantage in real-world problems. In addition, to better understand the true positive detection sensitivity of scBalance for each cell type, we then analyzed the precision of scBalance in these three datasets (Supplementary Tables 1–3). The results show that scBalance is the most robust and sensitive method for identifying the minor cell types compared with the other methods, especially under the complex cell background.

In summary, scBalance performs well on the baseline annotation task, as it has the stable ability to not only successfully identify the major cell types but also the minor cell types.

### scBalance outperforms in rare population identification in the inter-protocol annotation task

**Fig. 3:**
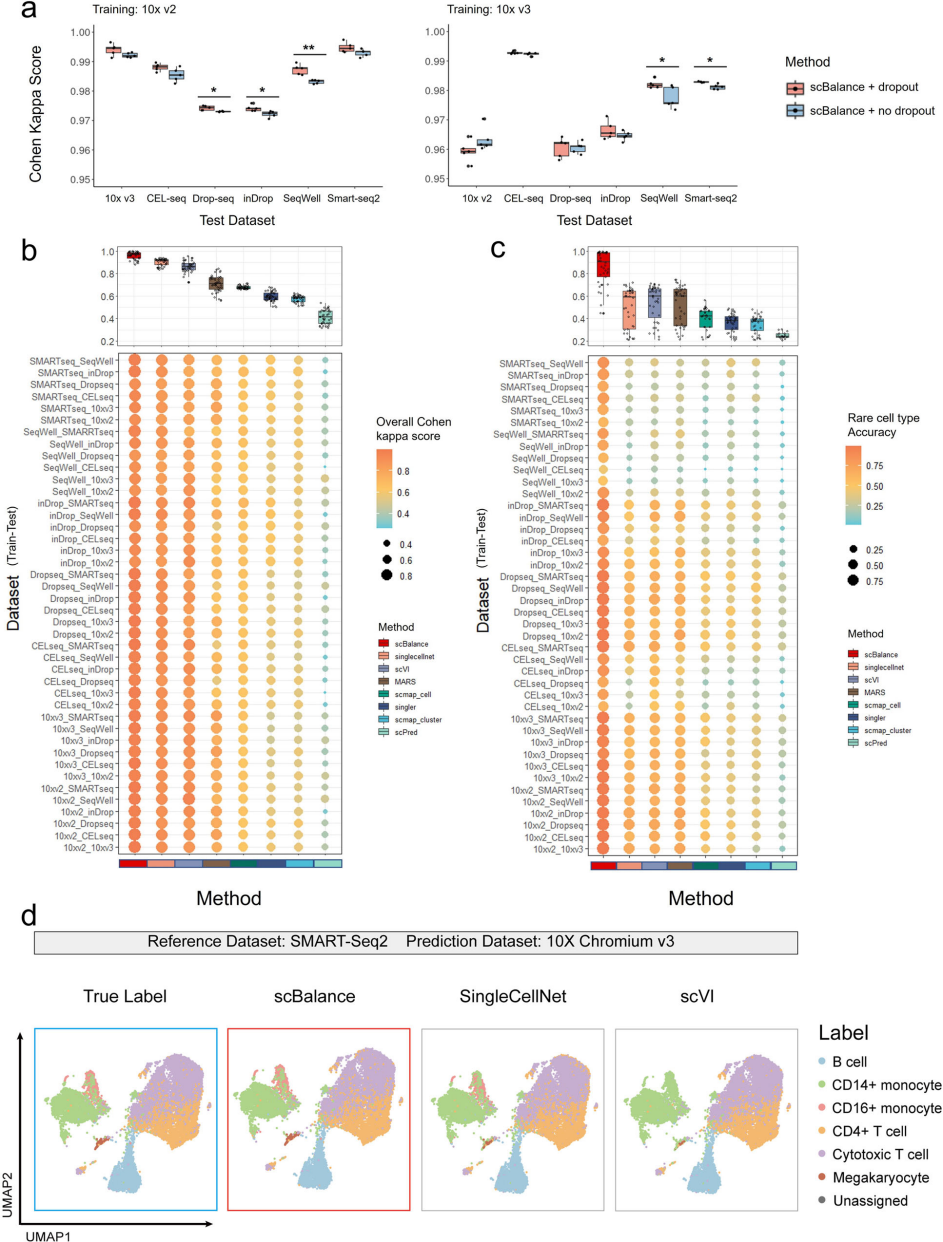
scBalance excels at identifying rare cell types in cross-platform annotation tasks. **a** Dropout technique is utilized in scBalance to enhance model generalization and robustness against noise. **b** scBalance’s overall annotation accuracy is compared to that of other methods on datasets generated by different protocols. Each experiment pair is named “Train Dataset_Test Dataset” and Cohen’s kappa score is used as the overall metric. (*n* = 42 for each boxplot to show all 42 training pairs). **c** scBalance’s ability to accurately identify rare cell types in inter-dataset annotation tasks is demonstrated. (*n* = 42 for each boxplot to show all 42 training pairs). **d** UMAP visualization shows that scBalance outperforms other methods in identifying rare cell populations across different approaches. All methods were trained on the PBMC dataset (SMART-Seq2) and used to predict cell types in the PBMC dataset (10xv3).

In the realistic scenario, it’s expected that users may train an annotation tool using a dataset that’s generated from a different protocol than the one used for the query scRNA-seq profile. However, when different sequencing platforms are used, more noise can be introduced, which can affect the inter-dataset annotation task more than the intra-dataset annotation task^38^. To improve the generalization ability of scBalance in cross-protocol tasks, we used the dropout technique to m`ake our model more robust to the technical variations. We first conducted a comparison experiment between scBalance with dropout and scBalance without dropout on the PBMCBench datasets from different sequencing platforms (Fig. 3a and Supplementary Fig. 5, and Supplementary Data 4) and the Pancreatic datasets from different protocols used in a previous study^39^ (Supplementary Fig. 6 and Supplementary Data 5). The results show that scBalance with dropout improves the generalization ability and leads to better performance in the inter-dataset annotation task for all sets of datasets. Moreover, we demonstrated the robustness of scBalance to batch effects in cross-dataset annotation tasks. We compared the classification performance of scBalance with and without batch correction using Combat^40^, a commonly used batch correction tool, to evaluate whether the performance of scBalance can be further improved by batch correction (Supplementary Fig. 7 and Supplementary Data 6). The results indicate that scBalance’s performance is not significantly impacted or improved by batch correction, suggesting that our method itself is robust to the potential negative effects of batch effects.

To further evaluate the performance of scBalance under batch effect and its ability to identify rare cell types, we expanded our benchmarking to include other annotation methods on the inter-dataset annotation task. We utilized the PBMCbench datasets (refer to “Methods” and Table 1) to test and evaluate the performance of each method on every protocol pair, with Cohen’s kappa score being used as the evaluation metric. Meanwhile, we were particularly interested in scBalance’s classification accuracy on minor cell populations, which we defined as cell types with less than 5% of the total cell number. Thus we also quantified the rare cell-type annotation ability along with the overall accuracy. The results, summarized in Fig. 3b, show that scBalance achieved the highest average scores across all experiments (Fig. 3b and Supplementary Data 7). Compared with the second-best method, scBalance elevated the average score from 0.85 to 0.95. Moreover, scBalance was also the best method on most of the test pairs, demonstrating its excellence on the inter-dataset task. Notably, we also analyzed the rare-type classification accuracy of each method (Fig. 3c), and the results show that scBalance outperforms the other methods in accurately identifying minor populations on most of the test pairs in the inter-dataset task. To further show the practicality and efficiency of scBalance, we conducted additional benchmarking experiments to evaluate its performance on the inter-dataset annotation task when other methods are used in conjunction with batch correction methods (Supplementary Fig. 8 and Supplementary Data 8). The results suggest that, while most of the methods demonstrated improvement (average improvements ranged from 1 to 4%) after batch effect correction preprocessing compared to Fig. 3b, scBalance continued to outperform the other methods for the inter-dataset annotation task. This indicates that scBalance remains one of the most efficient tools available for this task. Subsequently, to gains further insights into the classification results of the rare cell population, we used Uniform Manifold Approximation and Projection (UMAP) to visualize the clustering result of the top three highest-performing methods with the prediction label or true label (Fig. 3d). Our analysis revealed that, compared with the true label, SingleCellNet displayed more incorrect annotations on the Megakaryocyte cells and CD16+ monocytes than scBalance. Similarly, scVI demonstrated more incorrect labels on the Megakaryocyte cells and even completely failed on the classification of CD16+ monocytes. In contrast, scBalance provided the most accurate annotation result on all six cell types and successfully labeled the two rare cell populations, Megakaryocytes and CD16+ monocytes. Taken together, the results indicate that scBalance offers a more robust performance than existing methods for cross-platform annotation tasks and retains its outstanding capability of identifying rare cell populations under the influence of technical variations.

### Fast and robustness on the running speed enhances the scalability of scBalance

**Fig. 4:**
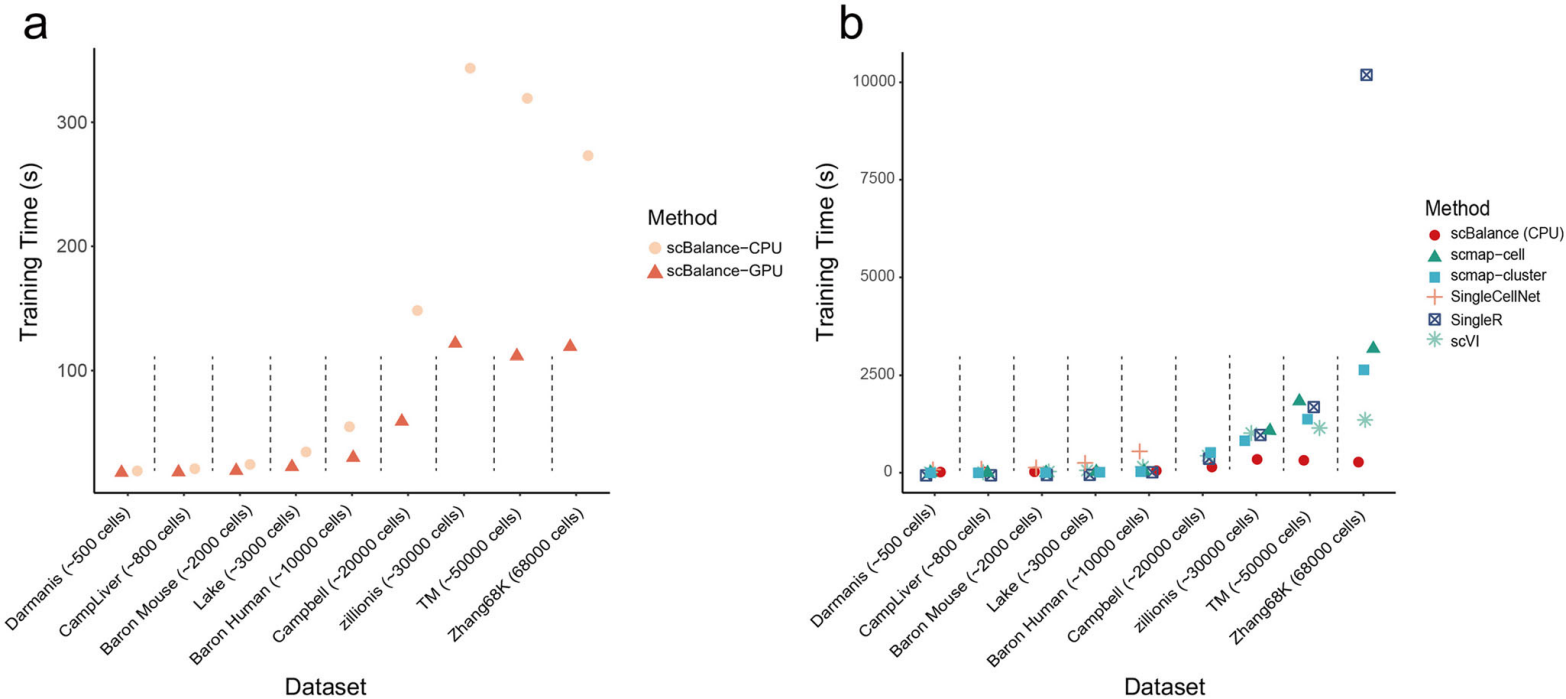
scBalance outperforms existing methods on speed and scalability. **a** Running time comparison of scBalance on datasets of different scales using different processors. Our method achieves fast running times with high scalability. **b** Comparison of the running times of six different methods on datasets of varying sizes. All methods are tested on the CPU. scBalance outperforms the other methods across all tested dataset sizes.

Running time is considered one of the most essential things for an annotation tool in the real single-cell analysis environment as well as the greatest obstacle to scalability. To highlight the superiority of the scBalance on the calculation speed, we presented the comparison results of the six representative methods which all have different basic machine-learning models (Fig. 4). Because of the usage of the GPU, we separately showed the scBalance-CPU and scBalance-GPU in order to make the comparison fair for other methods without GPU computation. We first compared the performance of the scBalance on the different processing units. The result indicates that scBalance-GPU has a large improvement in the running speed, which reduces more than 50% running time compared to the scBalance-CPU (Fig. 4a). Especially, scBalance-GPU gives a robust performance on the datasets with different cell numbers. The running time keeps relatively stable on the samples from 30k cells to 60k cells. This robustness gives scBalance a potential expanding ability to annotate large-scale datasets in a fast manner. We also presented the comparison result of scBalance-CPU with the other five methods. Even though all the methods are based on the CPU, scBalance also gives a promising running speed. Notably, in the datasets with more than 30k cells, scBalance reduces the running time to 10% of the other five methods. In the largest dataset, scBalance gives more than 20 times the computation speed compared with SingleR (Fig. 4b). The advantage in time-consuming also makes scBalance an outstanding tool in large-scale dataset annotation.

### Revealing bronchoalveolar immune cell atlas in COVID patient proofs the scalability of scBalance

**Fig. 5:**
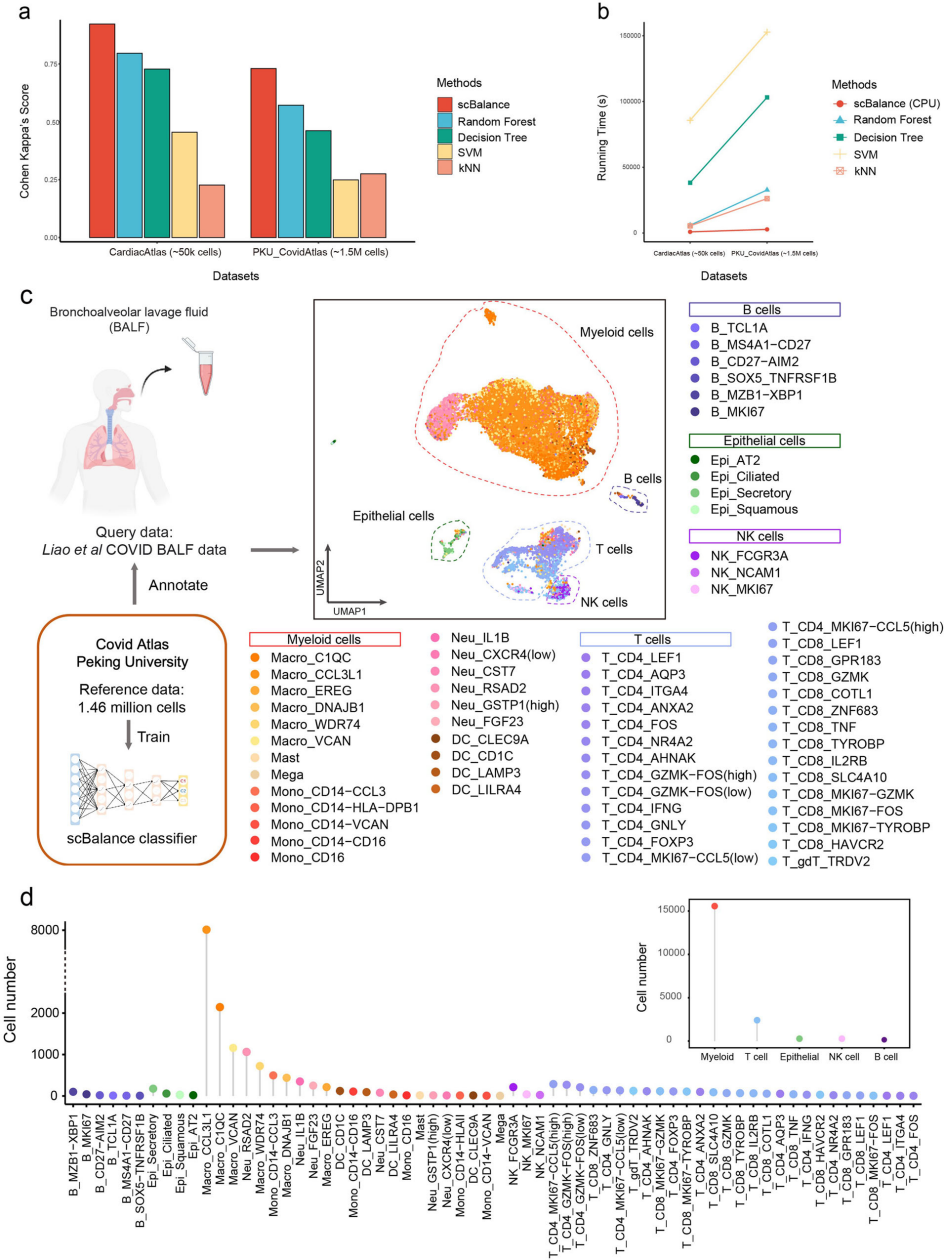
scBalance shows scalability by revealing immune landscape of BALF cells. **a** annotation performances compared with different methods on Cardiac Atlas (~50 K cells) and COVID Atlas (~1.5 M cells). **b** Running time comparison between scBalance and traditional machine-learning algorithms. *Y* axis shows running time in second. **c** UMAP shows the annotation result of scBalance. The reference dataset is COVID Atlas^17^ and the query dataset is BALF data^32^. **d** Dotplot shows the cell subtype distribution in the BALF dataset.

As the size of the cell atlas continues to increase, the scalability of annotation tools becomes more important. We thus discussed the strength of scBalance to learn rare cell types in the million-level scRNA-seq datasets. We first used the intra-dataset annotation result as proof of concept to evaluate the annotation performance of scBalance on the large-scale cell atlas. We collected two recently published cell atlas including human heart cell atlas^41^ (487,106 cells) and COVID-19 immune atlas^17^ (1,462,702 cells). As no other existing methods have reported annotation ability on million-level scRNA-seq profiles, especially it is even hard to load the dataset for R-based methods such as SingleCellNet and Scmap, we compared scBalacne with conventional machine-learning methods such as random forest (n_estimators=50,random_state=10), decision tree, SVM (kernel:rbf), and kNN (*k* = 3) in Python. As shown in Fig. 5a and Supplementary Data 9, scBalance significantly outperforms the other machine-learning methods on both two cell atlases. In addition, compared with the other methods, scBalance achieves up to 150 times faster running speed when training and labeling the COVID cell atlas (Fig. 5b). Even the threefold increase in cell number between the two datasets, scBalance remains the only method with a robust running speed, providing an advantage in scalability.

In addition to the simple evaluation of the scalability, we used COVID immune atlas as the reference dataset for an instance to illustrate that the annotation result of scBalance can effectively identify rare cell types when training with million-scale references. We also collected Bronchoalveolar lavage fluid (BALF) cells scRNA-seq profile from a severe COVID patient as the query data (Fig. 5c). While there are lots of publications discussing PBMC landscape^42–45^ in different COVID patient samples, the BALF cell component of COVID patients still lacks investigation. But as the sample that can most directly reflect microenvironment information on lung alveoli, BALF cells are of great importance to understanding the association of the disease severity and respiratory immune characteristics dynamic. Although Liao et al. revealed bronchoalveolar immune cells landscape in patients with COVID in 2020^32^, their work which is based on the integration of Seurat only identified cell groups in a low resolution. Here, we used scBalance to annotate BALF scRNA-seq dataset. Our method successfully identified much more cell subtypes than the original research by using the COVID atlas as the reference. Compared to the manual labeling method used in the original analysis, scBalance significantly improved annotation resolution for the BALF dataset. In combination with the result in Fig. 5c, d and Supplementary Fig. 9, scBalance identified 64 subtypes of the immune cells in the BALF sample. As expected, macrophages show the highest enrichment in the BALF sample whereas B cells only be a small part of the immune landscape. Notably, scBalance also identified rare subtypes in all cell groups. In the myeloid group, our method elucidates that there are also monocyte locates in the BALF instead of only macrophages. But macrophage cells are still the major component, especially the pro-inflammatory macrophage (M1) such as CCL3L1^+^ macrophage, which suggests a strong immune cell recruitment signal in BALF in the severe patient. Meanwhile, different from the analysis by Liao et al.^32^, our method reveals that the pro-inflammatory environment is not only produced by macrophages but also by CD14 monocyte (CCL3^+^). Furthermore, our method also found that a significant expansion of proliferative memory T cells (including MKI67-CCL4 (high) CD4 T cell and MKI67-CCL4 (low) CD4 T cell), compared with effector T cells, are enriched in the lung region. Together, our methods successfully identified cell subtypes and provide a more comprehensive immune atlas in the BALF by using the COVID cell atlas as the reference. It is worth noting that most of the cell types revealed by scBalance are rare in the COVID atlas, which further presents the advantage of identifying rare cell types of our method in the large-scale scRNA-seq dataset.

## Discussion

Recent advances in scRNA-seq methods have led to a growing need for cell-type annotation tools. As more well-defined cell atlases are published, auto-annotation tools are becoming increasingly popular. However, limitations in current software exist in the areas of rare cell-type labeling, scalability, and compatibility. In this article, we present scBalance, an open-source Python package that integrates adaptive weight sampling and a sparse neural network for supervised cell-type auto-annotation. We have demonstrated scBalance’s rare-type annotation ability and superior overall cell annotation ability through intra- and inter-dataset comparison experiments on several scRNA-seq datasets of different scales, generation protocols, and degrees of imbalance. Notably, Compared to most of the widely used cell-type annotation tools^14,30,31^, scBalance has shown excellent rare cell-type annotation ability, even in large datasets with complex cell backgrounds, where other methods fail to identify minor populations. In addition, we have demonstrated the robust running speed of scBalance on datasets of various scales, giving it a potential advantage for scalability. By testing our method on two recently published large cell atlases, we have further demonstrated scBalance’s scalability and rare population identification capacity in million-scale datasets. By utilizing this ability, scBalance has successfully described an immune landscape of BALF cells and identified more rare types than published research. Moreover, scBalance is designed to be compatible with Scanpy and Anndata, providing a user-friendly application.

In addition to introducing our method, we also show how scBalance can work with other software to offer users a broader range of applications. As illustrated in Fig. 1 and the GitHub tutorial, we provide optional parameters for users to use an external cell-type balancing method such as scSynO^34^ to better focus on a specific minor cell type of interest. We believe that incorporating these complementary tools into our method can significantly improve the performance of scBalance on various types of tasks, which could further expand the potential user population of scBalance.

Finally, we suggest several future efforts to improve scBalance, for example, including more prior knowledge such as marker genes to make more accurate annotations for similar cell types, such as CD4 + /CD45+ naïve T cells and CD4 + /CD45+ memory T cells. In addition, scBalance could be modified to annotate single-cell chromatin accessibility sequencing (scATAC-seq) data by adjusting the network to a sparse-robust structure. In summary, we believe that scBalance is a valuable addition to the auto-annotation toolbox, especially due to its rare cell-type annotation ability and scalability.

## Methods

### Datasets

In this section, we will describe all the datasets we used in the experiments and analysis above. In the baseline annotation experiments (intra- and inter-dataset), we used 20 datasets from small scale (~200 cells) to large scale (~70k cells). To further demonstrate the generalization ability of scBalance, all the selected datasets are generated from different complexities and different sequencing protocols. In the scalability experiments, two ultra-large datasets are used. All the datasets and their corresponding cell-type labels are obtained from the original paper. Corresponding details are shown in Table 1.

### scBalance pipeline

We provide scBalance, a compounded neural network structure, to conduct cell-type annotation tasks. scBalance requires a single-cell RNA expression matrix M as an input, in which each column represents a gene, and each row represents a cell. To obtain a more accurate annotation result, we recommend using a filtered dataset with log transformation and normalization as the training set. Log transformation and normalization steps can follow the standard preprocessing pipeline in the Scanpy tutorial. The goal is to prevent the outlier genes from interfering training process. Preprocessing can be done by following the tutorial of Scanpy, in which the scale parameter can be manually changed in the normalization function. The prediction dataset should have the same preprocessing steps as the training set. Before training, subsets will be extracted from the reference set and predicting set based on the common genes and be used as the input. scBalance pipeline consists of three core modules (Fig. 1a), a weighted sampling function and a neural network classifier.

### Weighted sampling function

The first module is a weighted sampling function that provides a simple but efficient solution for the learning imbalanced scRNA-seq datasets. Unlike commonly used oversampling and under-sampling methods, scBalance offers a combination of these two methods, thus significantly improving running speed without overfitting the minor types. In the training step, because we have the known labels in the training set, scBalance gives a weight to each cell type according to the proportion and randomly chooses samples from the dataset based on the weights to construct the training batch for the neural network. The sampling process is set with replacement to ensure the classifier can learn as much as possible minor type information in a reliable way.

### Neural network classifier

In the second module, we used a neural network (NN) structure to conduct the classification task. The NN classifier in scBalance contains an input layer, three hidden layers, and a softmax layer. The number of neurons in the input layer equals the number of genes in the scRNA-seq dataset. Following the three hidden layers have 256, 128, and 64 units, respectively. We also add dropout and batch normalization techniques at each hidden layer to overcome overfitting and increase running speed. Only the training stage of scBalance involves forward propagation with Batch Normalization and Dropout techniques. To avoid the variance shift^46^, we put the Dropout layer after the Batch Normalization layer (Eqs. (1–4)):

1 $${x}^{l-1}={BN}({x}^{l-1})$$

2 $${x}_{j}^{l}=\sigma (\,{W}_{j}^{l}{x}^{l-1}+{b}^{l})$$

3 $${r}^{l} \sim {Bernoulli}\,\left(p\right)$$

4 $${\widetilde{x}}^{l}={r}^{l}{x}^{l}$$

where *l* represents the *l*th layer of the neural network, *j* represents the *j*th neuron in its layer, b represents the random bias added in the layer, and $\sigma (\bullet )$ represents activation function. ${BN}(\bullet )$ is the batch normalization function to normalize the value of each mini-batch. *r* is a vector of independent Bernoulli random variable with the dropout probability *p*. This vector multiplied element-wise with each hidden layer to create dropout layer ${\widetilde{x}}^{l}$. In scBalance, the default dropout probability is 0.5. The activation function (Eq. (5)) in scBalance is exponential linear unit (ELU) function,

5 $$f\left(x\right)=\left\{\begin{array}{l}\qquad\quad\;\; x,\,x\ge 0\\ \!\!\alpha e({e}^{x}-1),\,x < 0\end{array}\right.$$

The output layer is based on the softmax function (Eq. (6)):

6 $$s\left({z}_{i}\right)=\frac{{e}^{{z}_{i}}}{{\sum }_{k=1}^{K}{e}^{{z}_{i}}}$$

where $z$ is the input vector of the softmax layer, *K* is the number of cell types in the reference dataset. In the backpropagation, we choose cross-entropy loss as the loss function of scBalance and the Adam^47^ optimization method as the optimizer. After training, the dropout layer will be disabled. scBalance provides a three-layer fully connected neural network for cell-type prediction.

### Hyperparameters

To demonstrate the effectiveness of the hyperparameters in scBalance, we compared different hyperparameter settings. (1) Activation function. In scBalance, due to the advantages of ELU in processing sparse datasets, we chose ELU as the activation. (2) Dropout layer. We then tested the performance of using the dropout layer. Because the dropout layer is designed mainly for batch effect, we design experiments following the cross-platform tasks. The result shows that using dropout layer improves the overall performance. Each value in the table comes from the average of five repeats.

### Software comparison and settings

To testify to the performance of scBalance, we compared it with several commonly used methods including R-based packages such as Scmap-cell, Scmap-cluster, SingleCellNet, SingleR, and scPred, and Python-based package scVI and MARS. All the evaluation codes and input data follow the instructions and tutorials provided by each package. To ensure our evaluation is fair to each method, we set all parameters as default for each approach, including scBalance.

The running environment we used for Python-based software is (1) scVI from Github (https://github.com/YosefLab/scvi-tools) version is 0.14.5. We ran the GPU version and set the hyperparameters following their example. We included LTMG inferring in preprocessing with the corresponding given option of the code. All the hyperparameters are set following the tutorial. The task is implemented on the workstation with Intel(R) Xeon(R) CPU E5-2667 v4, CentOS Linux release 7.7.1908 operation system, Nvidia TITAN X GPU, and 503GB physical memory. (2) MARS from Github (https://github.com/snap-stanford/mars). All the hyperparameters are set following the tutorial. The task is implemented on the server Linux Ubuntu 20.04.4 with 2.35 GHz AMD EPYC 7452 32-Core Processor and 503 G RAM. For the R-based packages, we implemented the tasks with the computer model Intel(R) Core(TM) i5-5287U CPU @ 2.90 GHz RAM 8GB. The details of the software are (3) SingleR version 1.6.1 from CRAN (https://github.com/dviraran/SingleR). The parameters are set as the default value provided by the tutorial. (5) Scmap-Cell and Scmap-Cluster from BioManager (https://github.com/hemberg-lab/scmap), with all parameters following the function instruction. For (5) scPred version 1.9.2 from BiocManager (https://github.com/powellgenomicslab/scPred), running with the default parameters. And (6) SingleCellNet version 0.1.1 from BiocManager (https://github.com/pcahan1/singleCellNet), running with the default parameters. We took the category with the largest score in the prediction to the final result. The task is implemented on the server Linux Ubuntu 20.04.4 with 2.35 GHz AMD EPYC 7452 32-Core Processor and 503 G RAM.

### Performance evaluation

We describe below the protocol and quantitative metrics we used in the experiments. To make the evolution reliable and able to quantify the variability, we used both fivefold cross-validation and 5-time repeating as the basic protocol in each of our experiments. For the fivefold cross-validation, the train-test split in the intra-dataset classification task is based on the StratifiedKFold function in sklearn v1.2.0 Python package. The split strategy is in a stratified fashion based on the ground truth label of the dataset. When testing, the true label of the test dataset will be hidden. The train-test split ratio is set as 0.8 (n_split=5) for all experiments in order to keep enough data in both the training set and the testing set. For the 5-time repeating test, the train-test split is based on the Train_test_split function in sklearn v1.2.0 Python Package. Random seed is applied to keep fairness. Each method will be tested five times. To evaluate the performance of the scBalance, we used Cohen’s kappa score, Macro F1 score and Accuracy in our paper. Cohen’s kappa score is for the overall performance metric. Unlike most of the papers which use Accuracy (Acc) as the metric, our aim is to testify to the identification ability of the rare cell types as well as the overall classification accuracy. Therefore, we choose Cohen’s kappa coefficient^48^ *k*, which is a minor-class sensitive approach thus can give us a comprehensive evaluation of classification performance, including the major types identification and the minor types identification (Eq. (7)),

7 $$k=\frac{{p}_{0}-{p}_{e}}{1-{p}_{e}}$$

where ${p}_{0}$ is the observed proportionate variable and ${p}_{e}$ is the hypothetical probability of chance variable. To calculate ${p}_{e}$, we use the observed data to calculate the probabilities of each observer randomly seeing each category. In this formula, the weight for misclassification of the rare populations will be highlighted.

Macro F1 score, because of its sensitivity to rare population, is used for the comparison of the sampling method (Eq. (8)).

8 $${Macro}\,F1=\frac{{sum}(F1\,{score})}{{number}\,{of}\,{classes}}$$

Accuracy is used to evaluate cell-type-specific accuracy in the intra-dataset annotation task and rare cell-type accuracy in the inter-dataset annotation task.

Precision is used as a true positive detection sensitivity metric (Eq. (9)):

9 $${Precision}=\frac{{TP}}{{TP}+{FP}}$$

In which TP is true positive and FP is false positive.

### Reporting summary

Further information on research design is available in the Nature Portfolio Reporting Summary linked to this article.

## Supplementary information

Supplementary Information

Description of Additional Supplementary Files

Supplementary Data 1-9

Reporting Summary

## Data Availability

No new data were generated for this study. All data used in this study are publicly available as previously described (see Table 1).

## Code availability

scBalance is available as an independent Python package at https://github.com/yuqcheng/scBalance.
