# Supplementary material for: A scalable sparse neural network framework for rare cell type annotation of single-cell transcriptome data
Source: Commun Biol. 2023 May 20;6:545. doi: 10.1038/s42003-023-04928-6 (PMC10199434; doi:10.1038/s42003-023-04928-6)
Supplement: Supplementary file 1 — Supplementary Information [file 42003_2023_4928_MOESM1_ESM.pdf]

Supplementary Information for:
A scalable sparse neural network framework for rare cell type annotation of single-cell transcriptome data

Yuqi Cheng[1,2], Xingyu Fan[3], Jianing Zhang[1], Yu Li[1,4,*]

[1]Department of Computer Science and Engineering (CSE), The Chinese University of Hong Kong (CUHK), Hong Kong SAR, China

[2]Weill Cornell Graduate School of Medical Sciences, Weill Cornell Medicine, New York, NY, 10065, USA

[3]School of Information and Software Engineering, University of Electronic Science and Technology of China, Chengdu, China, 610054, China

[4]The CUHK Shenzhen Research Institute, Hi-Tech Park, Nanshan, Shenzhen, 518057, China.

Contents:

Supplementary Figure 1: Balancing method evaluation based on the Baron Mouse dataset. 3

Supplementary Figure 2: Running time cost and memory space consuming evaluation among different sampling techniques. 4

Supplementary Figure 3: Cell-type-specific classification result on the Muraro dataset. 5

Supplementary Figure 4: Cell-type-specific classification result on the Zheng 68K dataset. 6

Supplementary Figure 5: Ablation study for the dropout layer on PBMCBench Datasets. 7

Supplementary Figure 6: Ablation study for the dropout layer on Pancreatic Datasets. 8

Supplementary Figure 7: Comparison between scBalance and scBalance + Combat 9

Supplementary Figure 8: Comparison between scBalance and the other methods + Combat in the cross-dataset annotation task. 10

Supplementary Figure 9: Violin plots show the selected marker genes for each identified cell type. 11

Supplementary Table 1: Averaged precision for each cell type in the Muraro dataset in 5-time repeating tests 12
Supplementary Table 2: Averaged precision for each cell type in the Baron Human dataset in 5-time repeating tests 13
Supplementary Table 3: Averaged precision for each cell type in the Zheng 68K dataset in 5-time repeating tests 14

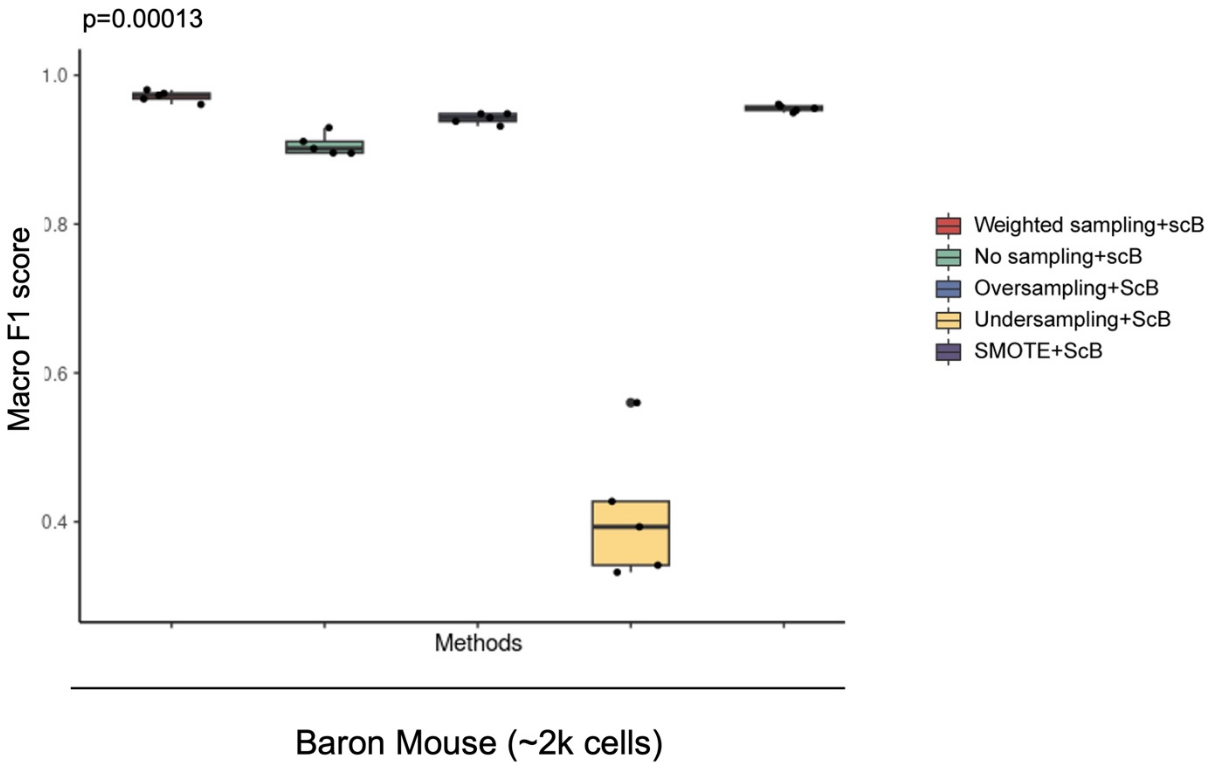

Supplementary Figure 1 | Balancing method evaluation based on the Baron Mouse dataset. n=5 for each boxplot.

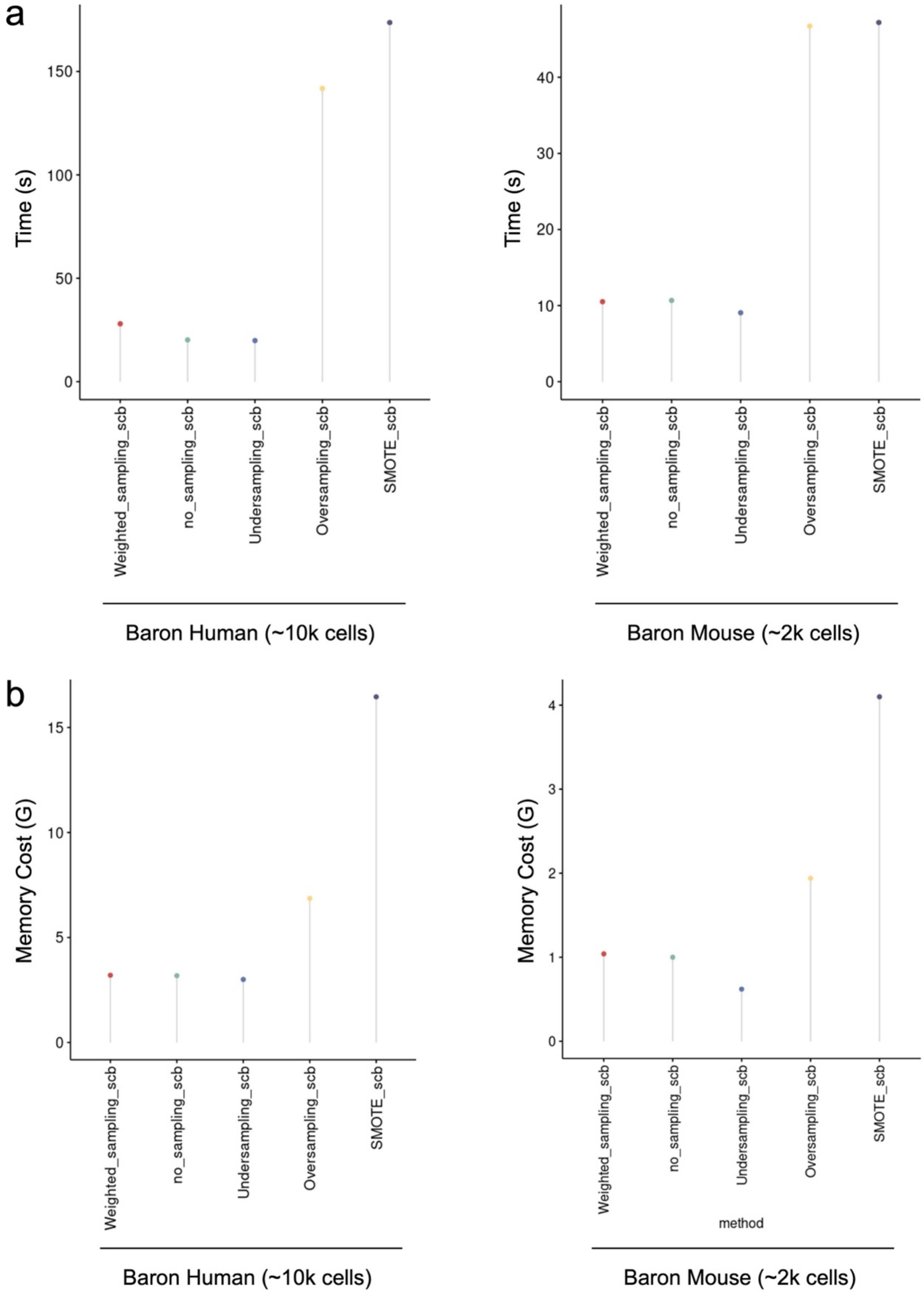

Supplementary Figure 2 | Running time cost and memory space consuming evaluation among different sampling techniques. a. Average running time. b. Average space consuming.

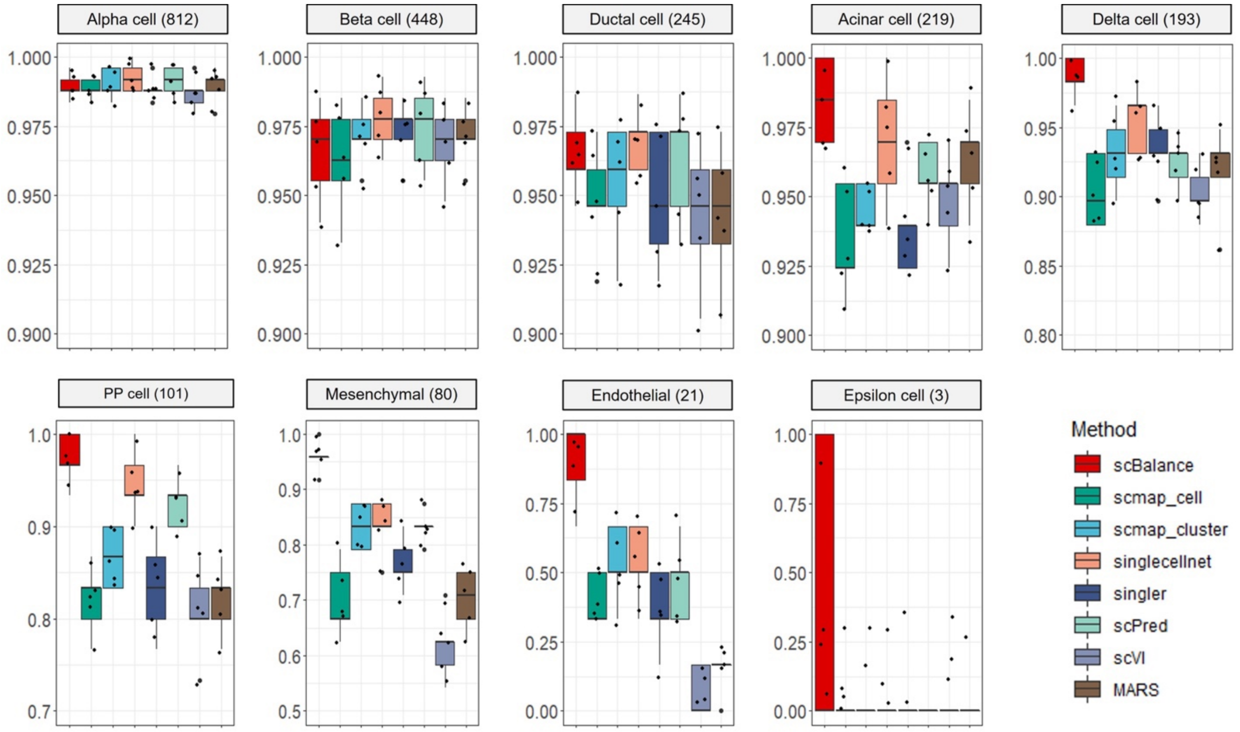

Supplementary Figure 3 | Cell-type-specific classification result on the Muraro dataset. The number following each cell type name is the number of cells in this cell type (n=5 for each boxplot).

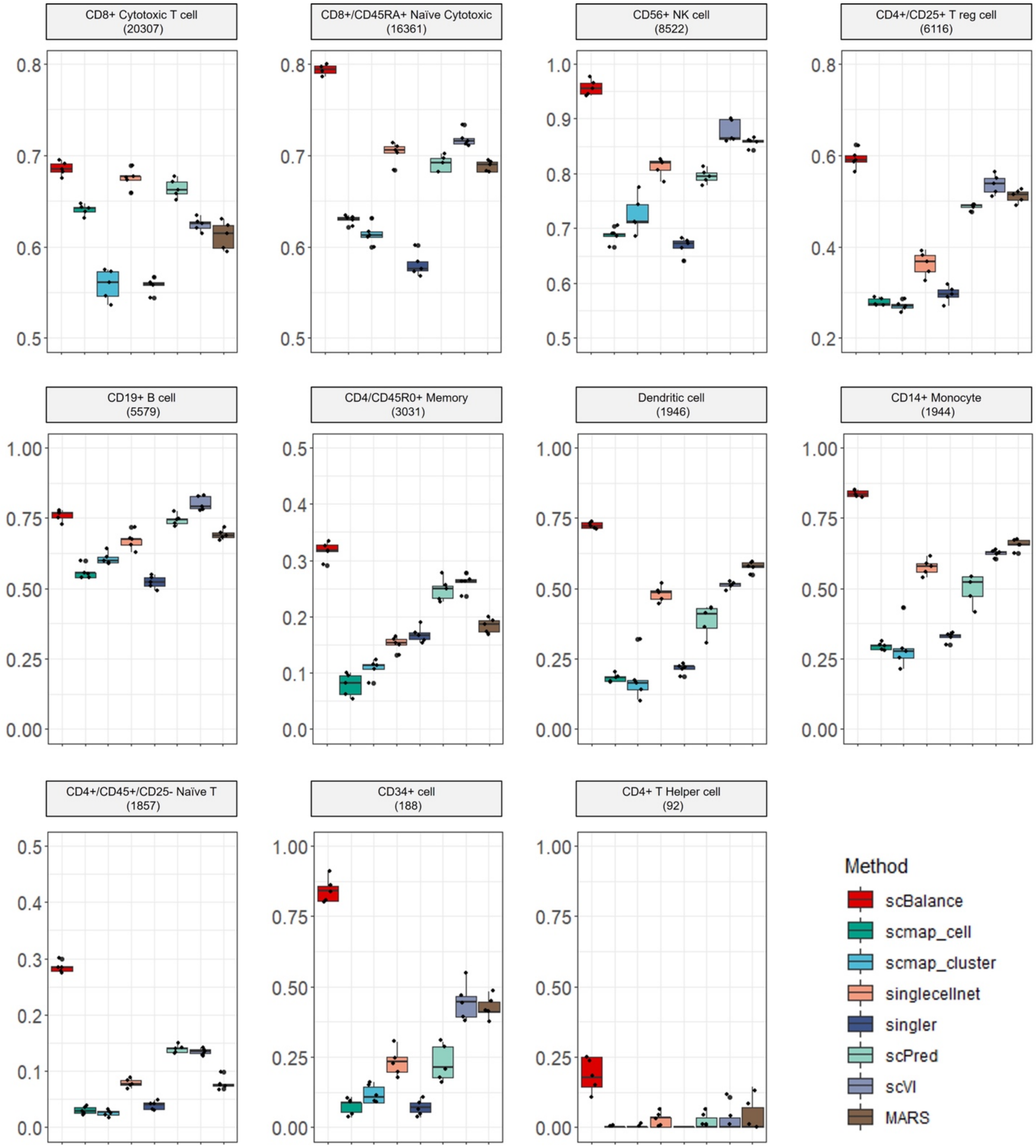

Supplementary Figure 4 | Cell-type-specific classification accuracy on the Zheng 68K dataset. The number following each cell type name is the number of cells in this cell type (n=5 for each boxplot).

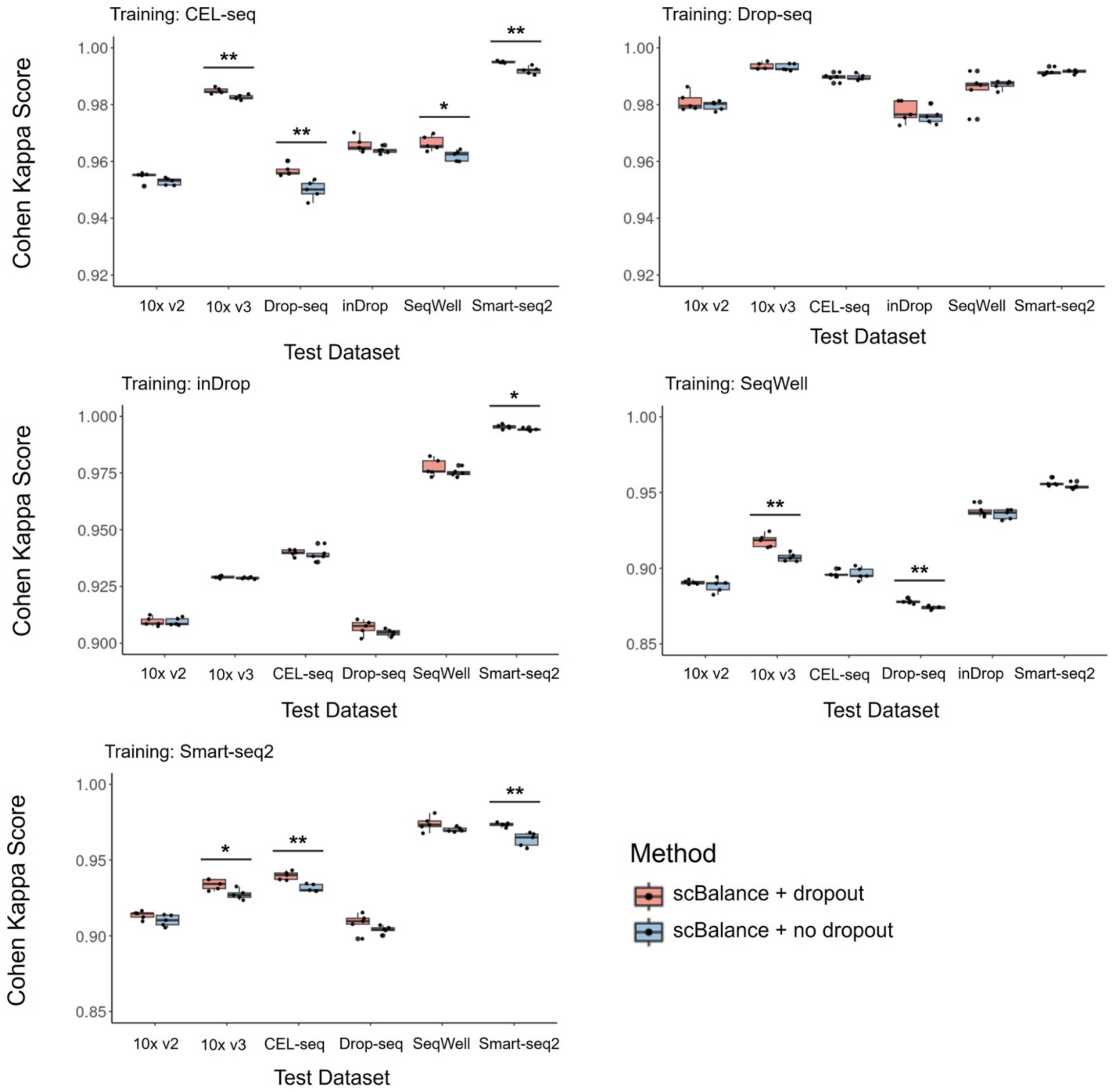

Supplementary Figure 5 | Ablation study for the dropout layer on PBMCBench Datasets. T-test. ns: non-significant, *:0.1, **:0.05. **:0.01. (n=5 for each boxplot).

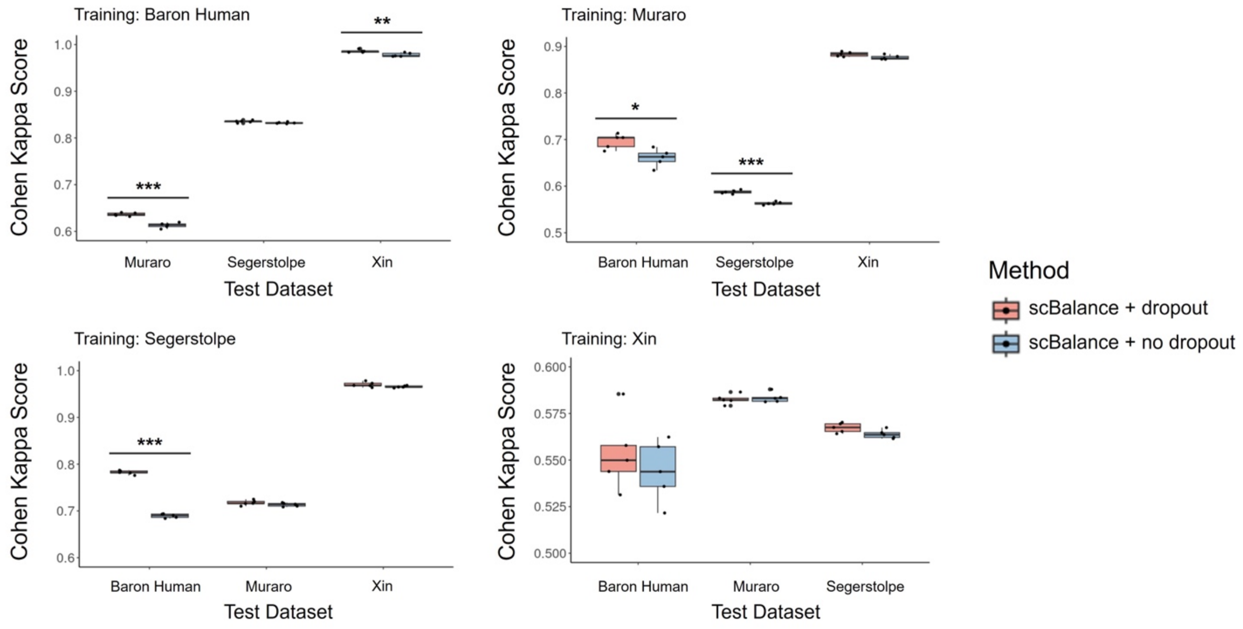

Supplementary Figure 6 | Ablation study for the dropout layer on Pancreatic Datasets. Significant test: T-test. ns: non-significant, *:0.1, **:0.05. **:0.01. (n=5 for each boxplot).

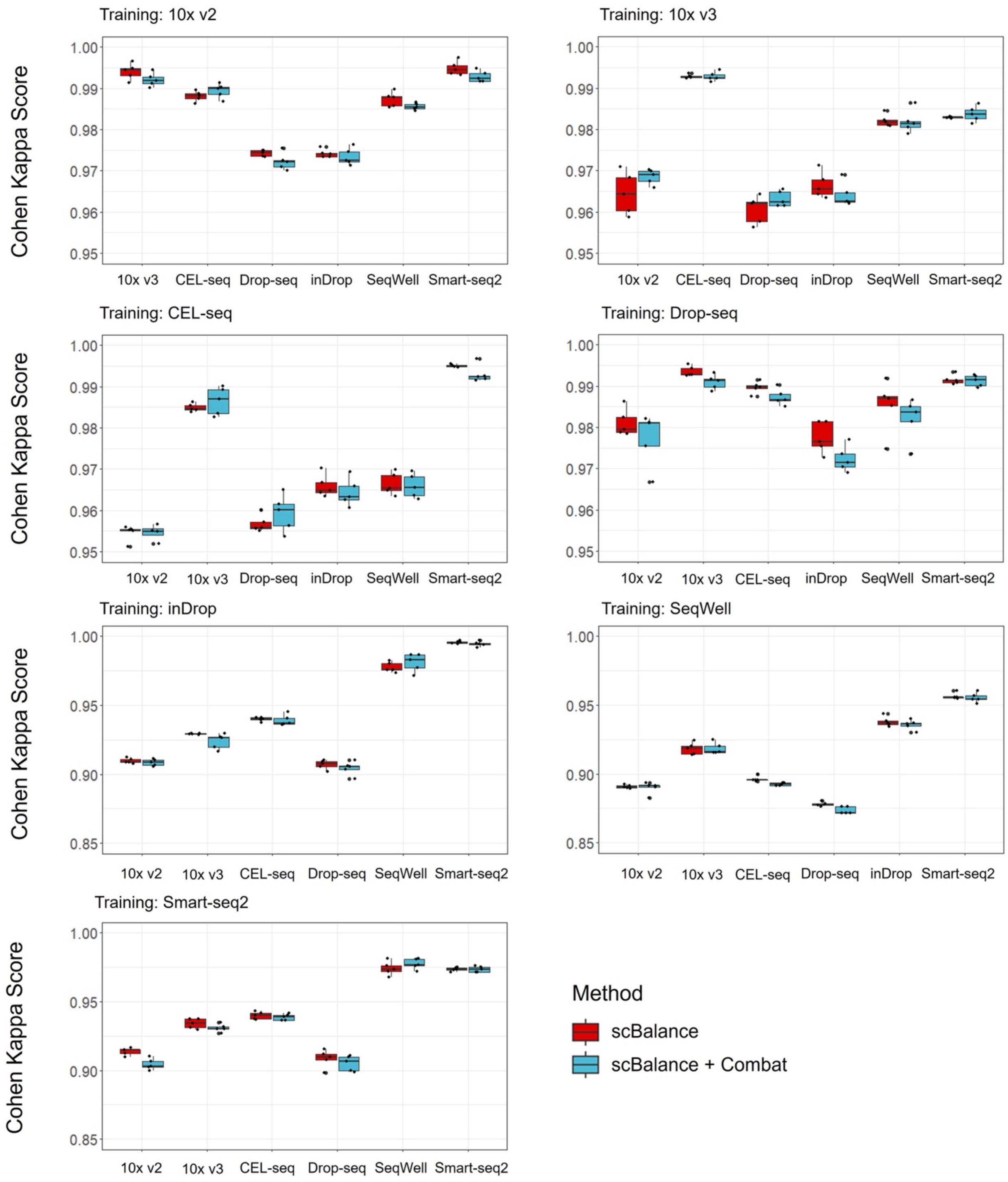

Supplementary Figure 7 | Comparison between with and without batch effect correction method in the cross-dataset annotation task. Significant test: T-test. ns: non-significant, *:0.1, **:0.05.   (n=5 for each boxplot).

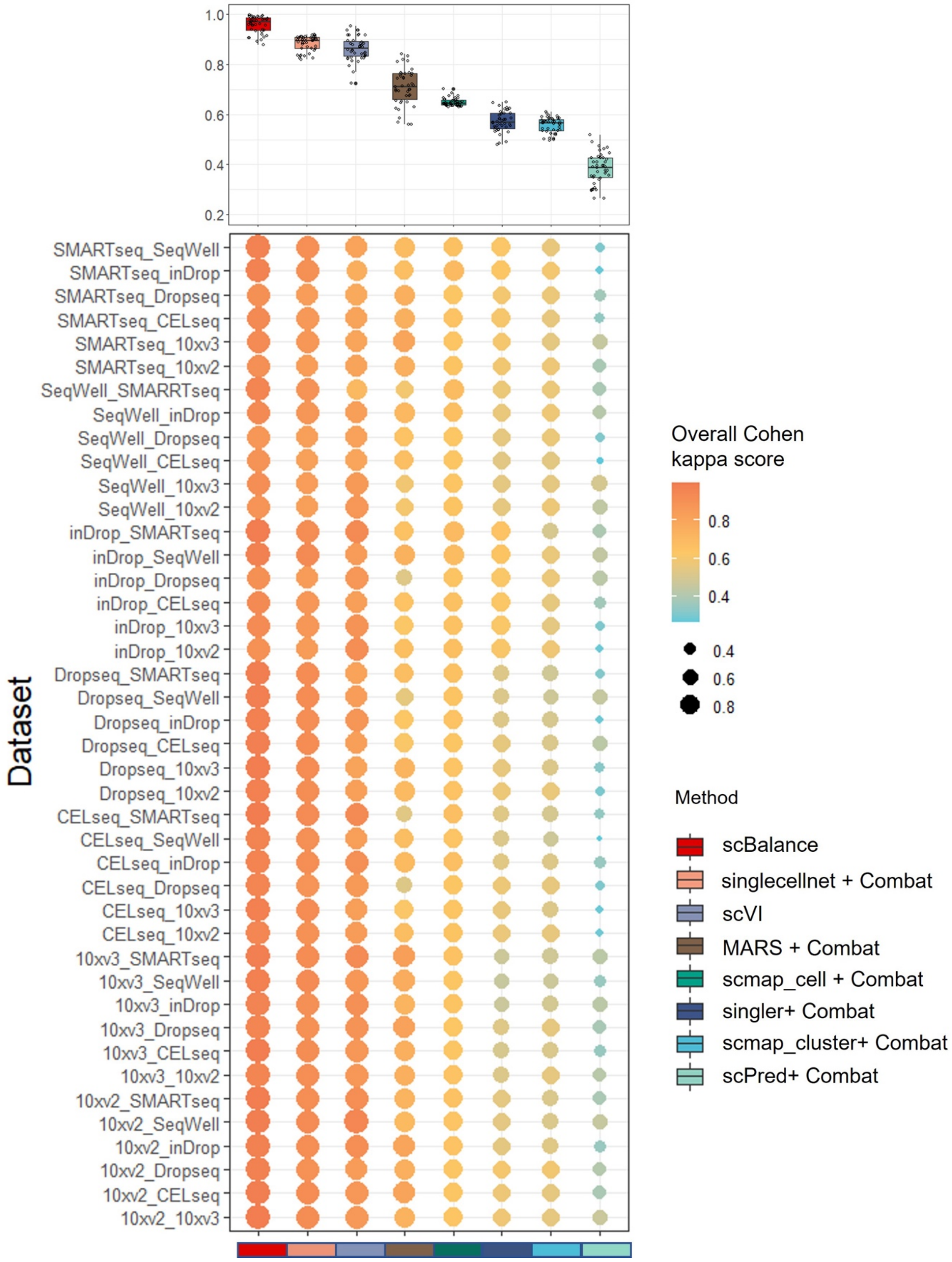

Supplementary Figure 8 | Comparison between scBalance and the other methods + Combat in the cross-dataset annotation task. n=42 for each boxplot to show all 42 training pairs.

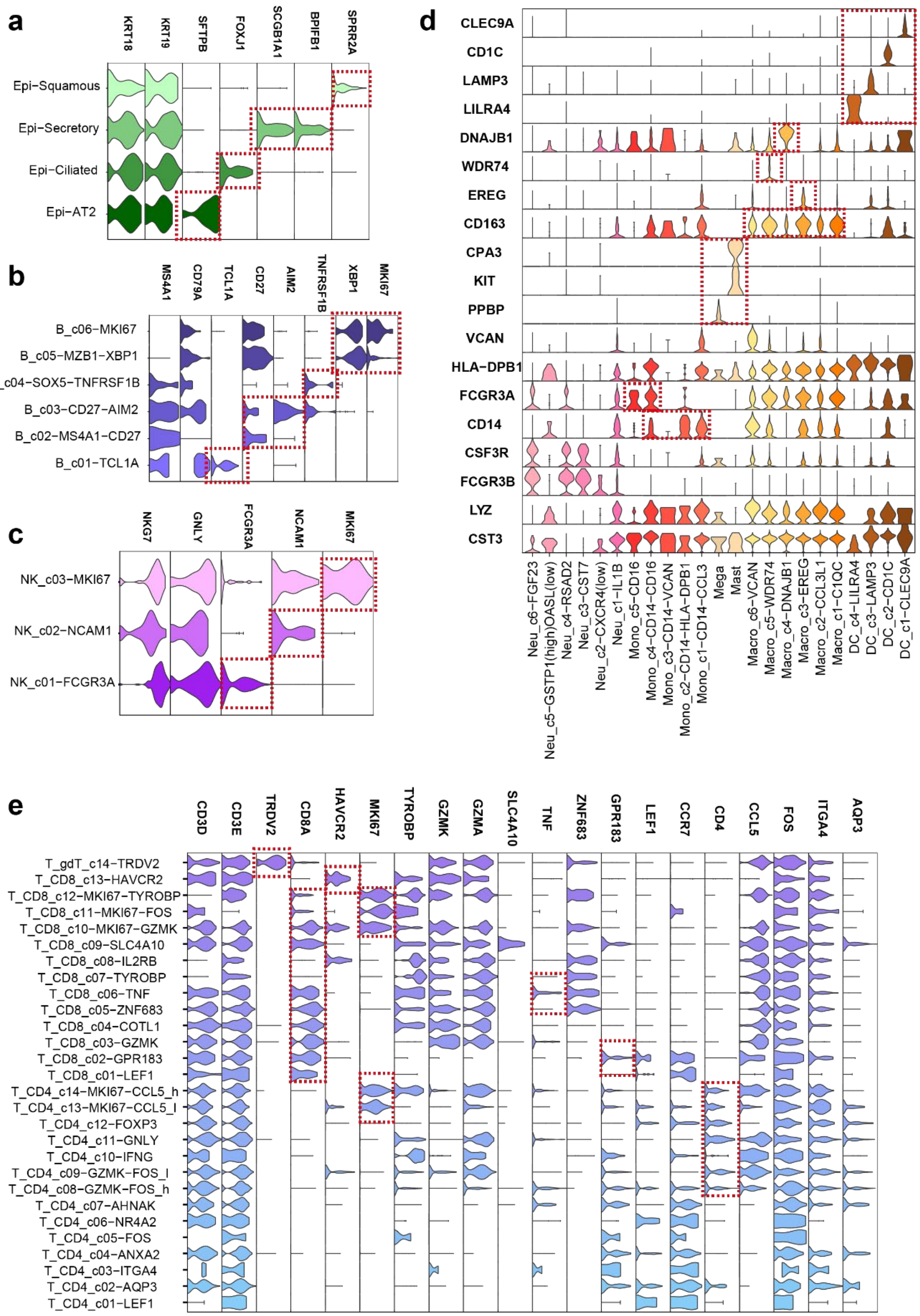

Supplementary Figure 9 | Violin plots show the selected marker genes for each identified cell type. Including **a.** 4 epithelial cell types, **b.** 6 B cell types, **c.** 3 NK cell types, **d.** 23 Myeloid cell types and **e.** 28 T cell types.

Precision	Alpha Cell	Beta Cell	Ductal Cell	Acinar Cell	Delta Cell	PP Cell	Mesenchymal	Endothelial	Epsilon Cell
scBalance	0.9958	0.9924	0.9951	0.9826	0.9902	0.9865	0.9877	0.9912	0.400
Scmap-cell	0.9893	0.9845	0.9649	0.9488	0.9414	0.8563	0.7452	0.5427	0.000
Scmap-cluster	0.9645	0.9568	0.9233	0.9476	0.9285	0.7469	0.7243	0.6728	0.000
SingleCellNet	0.9959	0.9936	0.9974	0.9843	0.9567	0.8944	0.8894	0.7289	0.000
SingleR	0.9875	0.9546	0.9645	0.9458	0.9235	0.7246	0.6498	0.2643	0.000
scPred	0.9917	0.9765	0.9783	0.9415	0.9436	0.8356	0.8076	0.3446	0.000
scVI	0.9958	0.9869	0.9802	0.9856	0.9841	0.9255	0.9344	0.8769	0.000
MARS	0.9711	0.9805	0.9479	0.9548	0.9623	0.9014	0.8547	0.4752	0.000

Supplementary Table 1 | Averaged precision for each cell type in the Muraro dataset in 5-time repeating tests. Each value is the average of 5 repeating. The red text shows the highest score in the corresponding cell type.

**Precision**	Beta Cell	Alpha Cell	Ductal Cell	Acinar Cell	Delta Cell	Activated Stellate	Gamma Cell
scBalance	0.9984	0.9965	0.9955	0.9967	0.9917	0.9975	0.9931
Scmap-cell	0.9945	0.9906	0.9879	0.9844	0.9146	0.8213	0.7682
Scmap-cluster	0.9978	0.9913	0.9893	0.9901	0.9384	0.8043	0.7168
SingleCellNet	0.9961	0.9970	0.9917	0.9889	0.9776	0.9416	0.9468
SingleR	0.9935	0.9879	0.9619	0.9622	0.9086	0.7567	0.7684
scPred	0.9989	0.9946	0.9805	0.9745	0.9546	0.8927	0.8904
scVI	0.9967	0.9942	0.9948	0.9950	0.9905	0.6786	0.8466
MARS	0.9814	0.9846	0.9628	0.9421	0.9078	0.8794	0.8396
**Precisionç**	Endothelial	Quiescent Stellate	Macrophage	Mast Cell	Epsilon Cell	Schwann Cell	T Cell
scBalance	0.9977	0.9893	0.9502	0.9131	0.8600	0.8834	0.6000
Scmap-cell	0.8456	0.4786	0.5728	0.3333	0.2000	0.0000	0.0000
Scmap-cluster	0.8764	0.6728	0.5837	0.3000	0.1000	0.2000	0.0000
SingleCellNet	0.9479	0.8047	0.7543	0.2833	0.3500	0.3000	0.0000
SingleR	0.8190	0.5372	0.2372	0.1200	0.0000	0.0000	0.0000
scPred	0.9372	0.7315	0.5125	0.4666	0.3667	0.2000	0.0000
scVI	0.7164	0.4683	0.4076	0.1000	0.0000	0.0000	0.2000
MARS	0.7048	0.4889	0.4134	0.2000	0.2000	0.0000	0.0000

Supplementary Table 2 | Averaged precision for each cell type in the Baron Human dataset in 5-time repeating tests. Each value is the average of 5 repeating. The red text shows the highest score in the corresponding cell type.

Precision	CD8+ Cyto T	CD8+/CD45RA+ Naive Cyto	CD56+ NK	CD4+/CD25+ T reg	CD4+ Cyto T	CD4+/CD45RA+ Memory
scBalance	0.6984	0.6873	0.8625	0.7628	0.7224	0.7763
Scmap-cell	0.5033	0.4758	0.6144	0.4331	0.4287	0.2638
Scmap-cluster	0.4708	0.4931	0.6837	0.2837	0.4729	0.1793
SingleCellNet	0.6172	0.5732	0.8374	0.6990	0.6438	0.3428
SingleR	0.4046	0.3897	0.4467	0.2438	0.4682	0.4158
scPred	0.5583	0.5779	0.8265	0.6672	0.6721	0.7004
scVI	0.5746	0.6044	0.7912	0.6731	0.6597	0.6825
MARS	0.5923	0.5134	0.8091	0.7046	0.7019	0.4799

Precision	Dendritic	CD14 monocyte	CD4+/CD45+/CD 25- Naive T	CD34+ Cell	CD4+ Helper T
scBalance	0.6951	0.7850	0.5477	0.7286	0.3895
Scmap-cell	0.2197	0.3768	0.1438	0.3200	0.0000
Scmap-cluster	0.1993	0.4095	0.1082	0.3728	0.0000
SingleCellNet	0.4761	0.6324	0.2836	0.4416	0.0000
SingleR	0.3332	0.3286	0.0975	0.2967	0.0000
scPred	0.4127	0.5972	0.1849	0.3989	0.0000
scVI	0.3864	0.4279	0.3408	0.5741	0.0667
MARS	0.4093	0.4488	0.2211	0.5962	0.1500

Supplementary Table 3 | Averaged precision for each cell type in the Zheng 68K dataset in 5-time repeating tests. Each value is the average of 5 repeating. The red text shows the highest score in the corresponding cell type.
