# Supplementary material for: A scalable sparse neural network framework for rare cell type annotation of single-cell transcriptome data
Source: Commun Biol. 2023 May 20;6:545. doi: 10.1038/s42003-023-04928-6 (PMC10199434; doi:10.1038/s42003-023-04928-6)
Supplement: Supplementary file 2 — Description of Additional Supplementary Files [file 42003_2023_4928_MOESM2_ESM.pdf]

Description of Additional Supplementary Files

File name: Supplementary Data 1
Description: Sampling method benchmarking results.

File name: Supplementary Data 2
Description: Cohen kappa score of the intra-dataset classification.

File name: Supplementary Data 3
Description: Cell type specific accuracy.

File name: Supplementary Data 4
Description: Dropout layer test (PBMC).

File name: Supplementary Data 5
Description: Dropout layer test (Pan dataset).

File name: Supplementary Data 6
Description: Reproduce data for Fig 3b.

File name: Supplementary Data 7
Description: Performance comparison on the intra-dataset annotation task for Extra-large dataset.

File name: Supplementary Data 8
Description: Batch effect robustness test (PBMC). All methods are used alongside Combat.

File name: Supplementary Data 9
Description: **s**cBalance/scBalance+combat.
