# Supplementary material for: A scalable sparse neural network framework for rare cell type annotation of single-cell transcriptome data
Source: Commun Biol. 2023 May 20;6:545. doi: 10.1038/s42003-023-04928-6 (PMC10199434; doi:10.1038/s42003-023-04928-6)
Supplement: Supplementary file 4 — Reporting Summary [file 42003_2023_4928_MOESM4_ESM.pdf]

nature portfolio

Corresponding author(s): Yu Li

Last updated by author(s): Apr 26, 2023

Reporting Summary

Nature Portfolio wishes to improve the reproducibility of the work that we publish. This form provides structure for consistency and transparency in reporting. For further information on Nature Portfolio policies, see our Editorial Policies and the Editorial Policy Checklist.

Statistics

For all statistical analyses, confirm that the following items are present in the figure legend, table legend, main text, or Methods section.

n/a	Confirmed	
☐	☒	The exact sample size (n) for each experimental group/condition, given as a discrete number and unit of measurement
☐	☒	A statement on whether measurements were taken from distinct samples or whether the same sample was measured repeatedly
☒	☐	The statistical test(s) used AND whether they are one- or two-sided<br>*Only common tests should be described solely by name; describe more complex techniques in the Methods section.*
☒	☐	A description of all covariates tested
☒	☐	A description of any assumptions or corrections, such as tests of normality and adjustment for multiple comparisons
☒	☐	A full description of the statistical parameters including central tendency (e.g. means) or other basic estimates (e.g. regression coefficient) AND variation (e.g. standard deviation) or associated estimates of uncertainty (e.g. confidence intervals)
☒	☐	For null hypothesis testing, the test statistic (e.g. F, t, r) with confidence intervals, effect sizes, degrees of freedom and P value noted<br>*Give P values as exact values whenever suitable.*
☒	☐	For Bayesian analysis, information on the choice of priors and Markov chain Monte Carlo settings
☒	☐	For hierarchical and complex designs, identification of the appropriate level for tests and full reporting of outcomes
☒	☐	Estimates of effect sizes (e.g. Cohen's d, Pearson's r), indicating how they were calculated

Our web collection on statistics for biologists contains articles on many of the points above.

Software and code

Policy information about availability of computer code

Data collection	The data is collected manually with the accession codes.
Data analysis	The running environment we used for Python-based software is (1) scVI from Github (https://github.com/YosefLab/scvi-tools) version is 0.14.5. We ran the GPU version and set the hyper-parameters following their example. We included LTMG inferring in preprocessing with the corresponding given option of the code. All the hyper-parameters are set following the tutorial. The task is implemented on the workstation with Intel(R) Xeon(R) CPU E5-2667 v4, CentOS Linux release 7.7.1908 operation system, Nvidia TITAN X GPU, and 503GB physical memory. (2) MARS from Github (https://github.com/snap-stanford/mars). All the hyper-parameters are set following the tutorial. The task is implemented on the server Linux Ubuntu 20.04.4 with 2.35GHz AMD EPYC 7452 32-Core Processor and 503G RAM. For the R-based packages, we implemented the tasks with the computer model Intel(R) Core(TM) i5-5287U CPU @ 2.90GHz RAM 8GB. The details of the software are (3) SingleR version 1.6.1 from CRAN (https://github.com/dviraran/SingleR). The parameters are set as the default value provided by the tutorial. (5) Scmap-Cell and Scmap-Cluster from BioManager (https://github.com/hemberg-lab/scmap), with all parameters following the function instruction. For (5) scPred version 1.9.2 from BiocManager (https://github.com/powellgenomicslab/scPred), running with the default parameters. And (6) SingleCellNet version 0.1.1 from BiocManager (https://github.com/pcahan1/singleCellNet), running with the default parameters. We took the category with the largest score in the prediction to the final result. The task is implemented on the server Linux Ubuntu 20.04.4 with 2.35GHz AMD EPYC 7452 32-Core Processor and 503G RAM.

For manuscripts utilizing custom algorithms or software that are central to the research but not yet described in published literature, software must be made available to editors and reviewers. We strongly encourage code deposition in a community repository (e.g. GitHub). See the Nature Portfolio guidelines for submitting code & software for further information.

Data

Policy information about availability of data

All manuscripts must include a data availability statement. This statement should provide the following information, where applicable:

- Accession codes, unique identifiers, or web links for publicly available datasets
- A description of any restrictions on data availability
- For clinical datasets or third party data, please ensure that the statement adheres to our policy

Deng dataset.(GSE45719) Muraro dataset.(GSE85241) Darmanis (GSE67835) Usoskin (GSE59739) CampLiver (GSE81252) Baron Mouse (GSE84133) Baron Human (GSE84133) Lake (phs000833.v3.p) Campbell (GSE93374) Zilionis (GSE127465) PBMCBench (https://doi.org/10.5281/zenodo.3357167) Tabula Muris Senis dataset.(https://figshare.com/projects/Tabula_Muris_Senis/64982) COVID large-scale dataset.(GSE158055) Cardiac Atlas (www.heartcellatlas.org) BALF dataset (GSE145926)

Field-specific reporting

Please select the one below that is the best fit for your research. If you are not sure, read the appropriate sections before making your selection.

☒ Life sciences ☐ Behavioural & social sciences ☐ Ecological, evolutionary & environmental sciences

For a reference copy of the document with all sections, see nature.com/documents/nr-reporting-summary-flat.pdf

Life sciences study design

All studies must disclose on these points even when the disclosure is negative.

Sample size	*Describe how sample size was determined, detailing any statistical methods used to predetermine sample size OR if no sample-size calculation was performed, describe how sample sizes were chosen and provide a rationale for why these sample sizes are sufficient.*
Data exclusions	*Describe any data exclusions. If no data were excluded from the analyses, state so OR if data were excluded, describe the exclusions and the rationale behind them, indicating whether exclusion criteria were pre-established.*
Replication	*Describe the measures taken to verify the reproducibility of the experimental findings. If all attempts at replication were successful, confirm this OR if there are any findings that were not replicated or cannot be reproduced, note this and describe why.*
Randomization	*Describe how samples/organisms/participants were allocated into experimental groups. If allocation was not random, describe how covariates were controlled OR if this is not relevant to your study, explain why.*
Blinding	*Describe whether the investigators were blinded to group allocation during data collection and/or analysis. If blinding was not possible, describe why OR explain why blinding was not relevant to your study.*

Reporting for specific materials, systems and methods

We require information from authors about some types of materials, experimental systems and methods used in many studies. Here, indicate whether each material, system or method listed is relevant to your study. If you are not sure if a list item applies to your research, read the appropriate section before selecting a response.

Materials & experimental systems

n/a	Involved in the study	
☒	☐	Antibodies
☒	☐	Eukaryotic cell lines
☒	☐	Palaeontology and archaeology
☒	☐	Animals and other organisms
☒	☐	Human research participants
☒	☐	Clinical data
☒	☐	Dual use research of concern

Methods

n/a	Involved in the study	
☒	☐	ChIP-seq
☒	☐	Flow cytometry
☒	☐	MRI-based neuroimaging
